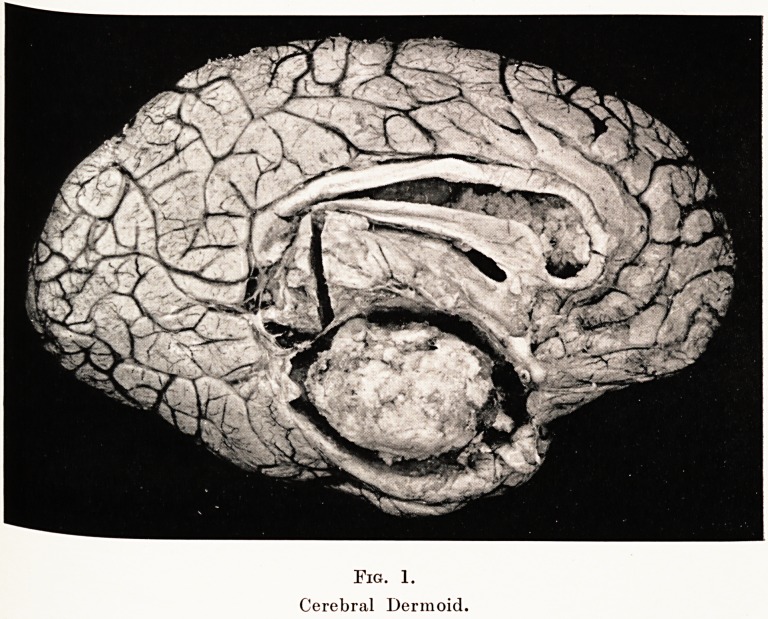# Cerebral Dermoid

**Published:** 1934

**Authors:** P. Phillips, D. M. Stone

**Affiliations:** Medical Superintendent, Southmead Hospital; Department of Preventive Medicine, Bristol University


### CEREBRAL DERMOID.

BY

P. Phillips, M.D., M.Sc.,
*Medical Superintendent, Southmead Hospital,*

AND

D. M. Stone, M.D., D.P.H.,
*Department of Preventive Medicine, Bristol University.*

---

Cerebral dermoids are of sufficient rarity to call for record in each instance.

The classical work on the subject is that of Bostroem,[1] published in 1897. More recently the subject of epidermoids and cholesteatomata has been dealt with by Percival Bailey and Horrax in America,[2] and by Critchley and Ferguson[3] in this country, but the rarer dermoids have received comparatively little attention. Brock and Klenke,[4] however, published in 1931 an account of a case of cerebral dermoid, and included in their paper a full and clear summary of all the recorded cases up to that date with a critical survey of their pathology.

The rarity of these growths is shown by the fact that until 1934 only 39 cases had been reported, and we have only been able to find details of three or four described since that time. In Cushing's series of 1,936 brain tumours only three were dermoids, and in the New York Neurological Institute a series of 450 verified tumours included but two dermoids.

Epidermoids are two or three times as common as dermoids, and according to Bostroem are embryonically older, arising in the first two or three weeks of fœtal life, whereas dermoids do not originate until the fourth or fifth week, when the skin has taken on a more definite form. The epidermoid is derived purely from ectodermal, the dermoid from ectodermal and mesodermal elements. The presence of hairs is, therefore, pathognomonic of dermoids.

In the 39 cases cited by Brock and Klenke 33 per cent. occurred in the posterior fossa, in the mid-line. The temporal lobe was the next commonest site (15 per cent.), and only 13 per cent. of all the growths communicated with the lateral ventrical. 28 per cent. of cases arose in the first decade, and 23 per cent. between 20 and 30 years of age; males predominated slightly; most of the growths were single and of pial origin.

The case we have recorded below is therefore typical, and its chief interest appears to us to lie in the extreme length of duration of symptoms and the gradual development of physical and mental changes.

The neoplasm was observed at the autopsy in a youth aged 25, who had been admitted to Southmead Hospital seven days previously comatose and cyanotic.

The patient was born in 1909, and attended Barley Field Elementary School. His mental condition first attracted attention at 12 years of age, when his case was reported for investigation in regard to his intelligence, with a view to his transfer to a Special School. On account of his age, however, no further steps were taken.

He left school at 14; his mother died a year later; he earned his living selling papers in the street. The money he earned he is said to have spent "in a silly manner." At 18 years of age he was admitted to Stapleton Institution and certified by Dr. Symes under the Mental Deficiency Act as follows: "Face asymmetrical, partial paralysis of right arm and leg. His memory is very poor. He failed on every occasion to repeat seven digits when dictated to him. Writes fairly, reads very imperfectly. Has difficulty in doing any simple calculation or problem in his head. Does not know the difference between a king and a president. He is good at problems of fact. Could not hold his own with other boys of his age on account of his physical and mental infirmities. He is subject to epileptic fits." His intelligence quotient by the Binet Simon scale was about sixty-two.

After his admission his mental condition slowly deteriorated, and he continued to have from fifteen to twenty fits per month, both "minor" and "major" in type. His general physique was good, robust, and well-nourished, but he was liable to acne, boils, and other septic conditions. On 15th March, 1934, he suddenly became exceedingly drowsy, and shortly after developed a squint and ptosis of the left eye. Examination of the discs showed bilateral optic neuritis. On 7th April, 1934, he was transferred to Southmead completely stuporose and somewhat cyanosed. Temperature 96·8°F. Pulse 120 per minute. Respirations 12 to 14 per minute. Examination of the eyes showed a complete ptosis on the left, and almost complete ophthalmoplegia of the left eye. The pupils did not respond to light and were unequal, the left being irregular and larger than the right. The right eye showed nystagmoid movements towards the mid-line. Both discs showed well-marked papillœdema. Pinching of the neck or tapping of the face on the left side resulted in a slow and limited spasm of the left facial muscles. Abdominal reflexes were absent. The right arm showed an old paralytic lesion with contractures; the left arm was freely movable; no tendon reflexes could be elicited either side. The legs showed partial paresis. The reflexes were present and normal. No abnormality was found in the other organs. Attempts at feeding were unsuccessful, save with a tube, and patient died on the 13th April.

*Pathology.*

At the post-mortem examination, carried out by one of us (P.P.), the right cerebral hemisphere and mid-brain showed no abnormality, and the right lateral ventricle, though distended by cerebro-spinal fluid, contained no foreign material.

The left hemisphere contained a large growth, the situation of which is delineated in the figure. The growth was originally enclosed within a thin membrane, so that actually it was a semi-solid cyst, but the membrane was torn on removing the brain. The temporal lobe was hollowed out on its superior and mesial aspects by the tumour mass, which compressed without invading the brain substance. The hippocampal gyrus and uncus were distinguishable but much distorted. The space formed measured about two and a half by two inches.

The growth consisted of :—

1. A solid, cream-coloured mass of cheesy appearance, fatty consistency, and friable texture. This filled the space above described, but was nowhere adherent to its walls. Scattered throughout its substance were numerous fine, short, silky hairs.

2. Small cauliflower-like masses of growth projecting from the walls of the space, adherent but easily removed.

3. Hairs, growing either singly or in masses ; some were fine, others were coarser and darker.

On opening up the left lateral ventricle this was found to be almost filled with the growth, extending into the anterior, posterior and descending horns. It was mainly of the warty, cauliflower-like type showing no marked evidence of compression. The masses of growth, inside the ventricle and without, connected up through a space at the tip of the inferior horn of the ventricle, apparently formed by a dilation of the lymphoid spaces around the choroid plexus of the lateral ventricle. This space was about 4 mm. in diameter, and was enclosed by a smooth-edged membrane. The brain substance and choroid plexus were free from neoplastic invasion.

The naked-eye appearance and the presence of dermal structures (hairs), together with its relation to the meninges, pointed to the growth being a true dermoid of pial origin—as opposed to the more frequently occurring epidermoid.

### PLATE VIII

FIG. 1.
Cerebral Dermoid.

*Histology.*

Sections taken across the brain tissue at the site where the growth of hair was thickest showed :—

1. Superficially a thin connective tissue layer containing well-formed blood vessels, small round cells, large cells with numerous central nuclei, hairs and hair follicles.

2. Deep to this an almost structureless and œdematous area, containing small round cells and devoid of nerve cells. Newly-formed vessels were present in its substance and one hair was embedded deeply in it.

3. The above area merged into a comparatively normal brain tissue, showing, however, some compression, with increase of neuroglia and round-celled infiltration.

Sections across the mass of growth showed :—

1. Areas of lipoidal material staining well with Scharlach R. and Nile Blue, patches of crystalline cholesterol deposits and myelin droplets.

2. Hairs with normal structure and slightly pigmented core.

3. Finely-fibrillated reticular or neuroglial tissue with associated cells.

4. Nerve cells of the ganglion type, containing granules and surrounded by small monocytes ; also nerve cells similar to those of the cortical cerebral layer.

5. Very many newly-formed blood-vessels, the walls consisting of a single layer of endothelial cells, and the width being that of a red blood cell only.

6. Large cells suggestive of an epidermal or dermal organ, situated mainly in relation to the hair follicles.

Owing to the absence of relatives it has been difficult to obtain adequate details of the patient's past history, and we are greatly indebted to Dr. Datta of Stapleton Institute and to Mr. A. C. J. Gregory of the Bristol Education Department for their help in this matter.

### REFERENCES.

[1] Bostroem, R., " Über die pialen Epidermoide, Dermoide, und Lipome und duralen Dermoide," *Centralbl. f. allg. Path. u. path. Anat.*, 1897, viii. 1.

[2] Horrax, G., " A consideration of the dermal versus the epidermal cholesteatomas, having their attachment in the cerebral envelopes," *Arch. Neur. and Psychiat,* 1927, viii. 265.

[3] Critchley, M., and Ferguson, F. R., "The cerebrospinal epidermoids (cholesteatomata)," *Brain,* 1928, li. 334.

[4] Brock, S., and Klenke, D. A., "A case of Dermoid Overlying the Cerebellar Vermis," *Bull. Neur. Inst. of New York,* 1931, i. 328.

## Figures and Tables

**Fig. 1. f1:**